# The target of rapamycin kinase is a positive regulator of plant fatty acid and lipid synthesis

**DOI:** 10.1093/plphys/kiae639

**Published:** 2024-12-02

**Authors:** Hui Liu, Jantana Blanford, Hai Shi, Jorg Schwender, John Shanklin, Zhiyang Zhai

**Affiliations:** Department of Biology, BNL 463, 50 Bell Ave, Upton, NY 11973, USA; Department of Biology, BNL 463, 50 Bell Ave, Upton, NY 11973, USA; Department of Biology, BNL 463, 50 Bell Ave, Upton, NY 11973, USA; Department of Biology, BNL 463, 50 Bell Ave, Upton, NY 11973, USA; Department of Biology, BNL 463, 50 Bell Ave, Upton, NY 11973, USA; Department of Biology, BNL 463, 50 Bell Ave, Upton, NY 11973, USA

## Abstract

In eukaryotes, target of rapamycin (TOR), a conserved protein sensor kinase, integrates diverse environmental cues, including growth factor signals, energy availability, and nutritional status, to direct cell growth. In plants, TOR is activated by light and sugars and regulates a wide range of cellular processes, including protein synthesis and metabolism. Fatty acid (FA) synthesis is a key to membrane biogenesis that is required for cell growth. To elucidate the primary regulatory role(s) of TOR in lipid metabolism, we followed FA and lipid changes in plants with altered TOR protein levels or activity for short durations, using *Nicotiana benthamiana* leaves, *Arabidopsis* seedlings, and *Brassica napus* cell suspension cultures. Transient expression of *TOR* significantly elevated the levels of total FA (TFA) in *N. benthamiana* leaves. Conversely, treatment of *Arabidopsis* seedlings with the TOR-specific inhibitor Torin 2 for 1 d caused significant reductions in FA and membrane lipids. Similarly, incubating oil–producing *B. napus* suspension culture cells with Torin 2 for 8 h led to significant decreases in the levels of TFA and triacylglycerol. The results from 3 independent systems presented here establish that TOR positively regulates lipid synthesis in plants, consistent with its role in animals. Furthermore, RNA-seq analysis of Torin 2-treated *Arabidopsis* seedlings showed that TOR promotes the upregulation of several genes involved in de novo FA synthesis while downregulating several genes involved in lipid turnover, which we propose as a mechanistic explanation for its promotion of lipid synthesis and accumulation.

## Teaser

The highly conserved TARGET OF RAPAMYCIN kinase positively regulates lipid synthesis in plant systems, consistent with its role in other organisms.

## Introduction

The target of rapamycin (TOR) is a central regulatory kinase found in all eukaryotic cells, serving as a central node in a network that controls cell growth by integrating information about the status of nutrients and energy to coordinate the synthesis or breakdown of cellular components. Once activated, TOR promotes cell growth by increasing both cell size and number (Xiong and Sheen 2014; Liu and Sabatini 2020). Growing and proliferating cells require enhanced lipid synthesis for the assembly of new membranes. In mammalian cells, the activated mammalian TOR complex 1 (mTORC1) promotes lipogenesis and at the same time inhibits lipolysis by activating the transcription factor sterol regulatory element binding protein 1/2 (SREBP1/2), the master regulator of sterol and fatty acid (FA) synthesis (Caron et al. 2015), and by suppressing the expression of adipose triglyceride lipase, which involves the action of the transcription factor early growth response 1 (Chakrabarti et al. 2010, 2013). Compared with the well-established roles of mTORC1 in promoting cell growth in mammals, our knowledge about the roles of TOR in plants, especially in lipid metabolism, is sparse. *Arabidopsis thaliana* possesses a single *TOR* gene coding for a protein of ∼280 kDa (John et al. 2011). Most of what we know comes from experiments involving prolonged suppression of *TOR* in *A. thaliana* using an estradiol-inducible artificial microRNA approach. Under these conditions, leaves accumulated increased levels of TAG with increased long-chain polyunsaturated FA composition (Caldana et al. 2013). This observation suggests that plant TOR may play a negative role in lipid accumulation, which seems inconsistent with the well-established role of mTORC1 in promoting lipid synthesis and storage in mammals and an early report that TOR downregulates gene expression of TAG lipase and acyl-CoA oxidase in *Arabidopsis* (Xiong et al. 2013). Like mTOR, plant TOR has been shown to negatively regulate autophagy, a degradation process for cellular components induced by stress conditions or during senescence (Liu and Bassham 2010, 2012; Li and Vierstra 2012). That the literature on the role of TOR in FA and lipid synthesis and accumulation remained incomplete and somewhat inconsistent motivated us to perform this study. Thus, here, we tracked the changes in total FA, membrane lipids, and storage lipids TAG using several independent plant systems in which the TOR protein level or its activity was disrupted for relatively short periods to address the primary role(s) of TOR in plant lipid metabolism. We observed that transient expression of *TOR* significantly elevated the total FA content (TFA) of *Nicotiana benthamiana* leaves. Consistent with this, short-term inhibition of TOR in *Arabidopsis* seedlings, either by estradiol-inducible gene silencing or by treatment with Torin 2, a TOR-specific inhibitor, significantly reduced both FA and lipid contents. Furthermore, Torin 2 treatment of a TAG-producing *Brassica napus* suspension cell culture significantly decreased the accumulation of both total FA (TFA) and TAG. Together, these data from 3 independent systems provide consistent support for TOR in promoting lipid synthesis. Additional support from RNA-seq analysis of the *Arabidopsis* seedlings following short-term TOR suppression suggests that, like mTOR in mammals, plant TOR primarily promotes lipid synthesis and storage while repressing lipid degradation.

## Results

### Transient expression of TOR in *N. benthamiana* leaves results in increased TFA levels

**Figure 1.**
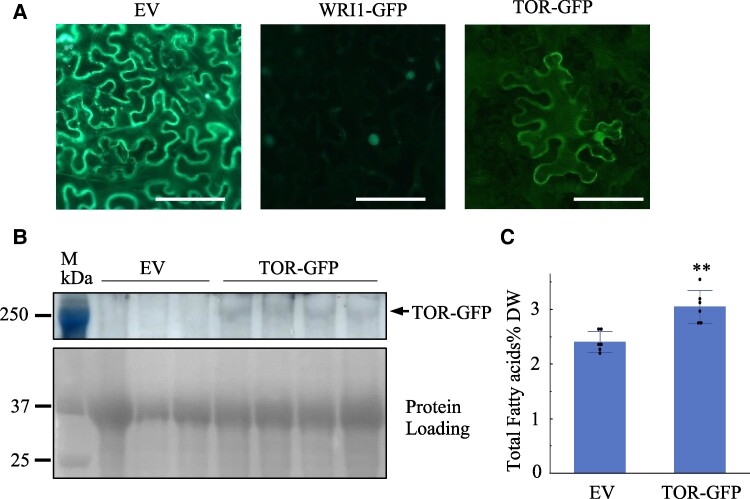
Transient expression of TOR in *N. benthamiana* leaves increases the levels of TFA. **A)** Representative fluorescence confocal image of *N. benthamiana* leaf samples taken 4 d after agroinfiltration with TOR-GFP. Bar = 50 *μ*m. **B)** Immunoblotting with anti-GFP antibody shows that TOR-GFP is detected in protein samples extracted from *N. benthamiana* leaves 4 d after infiltration with agrobacterium containing plasmid TOR/pCHF3-hGFP (TOR-GFP), but not pCHF3-hGFP (empty vector [EV]). Ponceau S staining of PVDF membrane after protein transfer is shown as a protein loading control. M, protein markers. Multiple lanes for the same construct represent biological replicates. **C)** Transient expression of TOR for 4 d after agroinfiltration significantly elevates TFA content in *N. benthamiana* leaves after. Bar values represent mean ± Sd (*n* = 6), with each data point represented by a dot. Asterisks denote a statistically significant difference from the EV (Student's *t*-test, **P < 0.01).

To explore the role of TOR in regulating plant lipid metabolism, we examined the effect of *TOR* overexpression on FA (the major component of lipids) content, 4 d after transient expression of a construct encoding a TOR-GFP fusion protein under the control of the 35S promoter in *N. benthamiana* leaves. Following infiltration of Agrobacterium, GFP fluorescence signal was detected in both the cytoplasm and the nucleus of cells (Fig. 1A), consistent with the previous reports of TOR's dual subcellular localization (Ren et al. 2011; Schepetilnikov et al. 2017). The successful expression of *TOR-GFP* was further confirmed by immunoblotting using a GFP antibody (Fig. 1B). Lipids were extracted from the parallel samples of *N. benthamiana* leaves, and TFA levels were subsequently analyzed. As shown in Fig. 1C, leaves expressing *TOR-GFP* exhibited a significant increase in TFA content, ∼27% higher than those infiltrated with the empty vector.

### Inducible gene silencing of TOR significantly reduces TFA content in *Arabidopsis*

**Figure 2.**
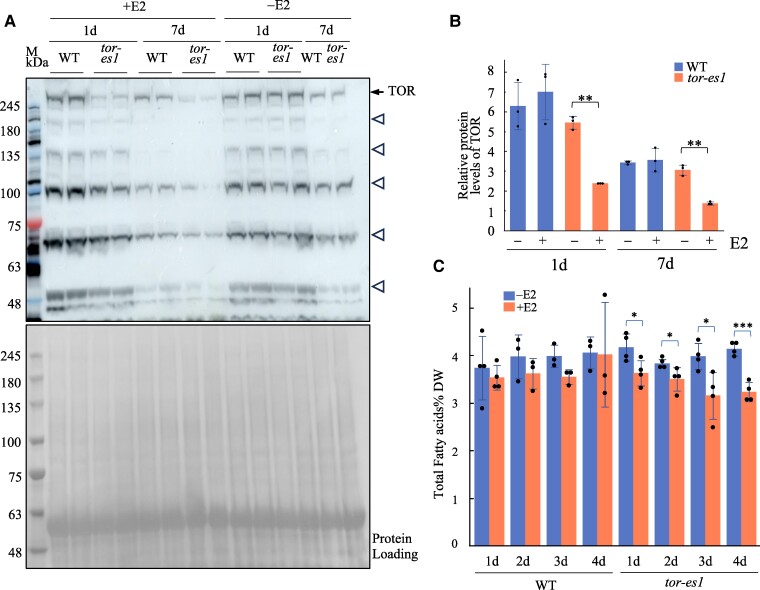
Inducible gene silencing of TOR significantly reduces the levels of TOR protein and TFA in *Arabidopsis.*  **A)** Representative immunoblot probed by custom TOR antibody shows TOR protein levels in *Arabidopsis* WT and *tor-es1* seedlings grown on ½ MS with (+) or without (−) supplementation of 1 *μ*m of estradiol (E2) for 1 or 7 d (top panel). Ponceau S staining of membrane after protein transfer is shown as a protein loading control (bottom panel). M, protein markers. The arrowheads indicate TOR (279 kDa). Additional signals indicated by open triangles may be fragments of the TOR resulted from proteolysis. **B)** Relative TOR protein levels in **A)** were quantified with GelAnalyzer2010 and normalized against corresponding protein loading. Data shown are mean ± Sd, *n* = 3 independent immunoblots; 2-tailed Student's *t*-test for comparison between *tor-es1* treated without E2 and with E2, **P < 0.01. **C)** TFA analysis in *Arabidopsis* WT and *tor-es1* seedlings grown in liquid medium (½ MS + 1% sucrose) supplemented without (−) or with (+) 1 *μ*m of E2 for 1, 2, 3, or 4 d. Bar values represent mean ± Sd (*n* = 4), with each data point represented by a dot. Asterisks denote a statistically significant difference from non-E2 treatment (Student's *t*-test, *P < 0.05; ***P < 0.001).

To assess the effect of TOR silencing on plant lipid metabolism, we employed a previously characterized *Arabidopsis* estradiol-inducible *tor* RNAi line (*tor-es1*; Xiong and Sheen 2012). A custom TOR antibody was generated to monitor native TOR protein levels after gene silencing. This custom TOR antibody was raised against a polypeptide corresponding to the C-terminal region of Arabidopsis TOR (amino acids 2282 to 2481) that was produced and purified from *E. coli* (Supplementary Fig. S1A). To confirm the specificity of the antibody for TOR, it was used in immunoprecipitation (IP) assays on protein extracts from 1-wk-old wild-type (WT) *Arabidopsis* seedlings. The IP samples were analyzed via SDS-PAGE, revealing a band above 250 kDa, which was confirmed as TOR by MS (Supplementary Fig. S1, B and C and File S1). Next, we tested the impact of estradiol (E2) treatment on TOR protein levels in *tor-es1*. Three-day-old WT or *tor-es1* seedlings grown on ½ MS medium were transferred into ½ MS alone or ½ MS supplemented with 1 *μ*m E2 for 1 or 7 d. Figure 2, A and B shows TOR protein levels in *tor-es1* were significantly reduced in the presence of E2 compared with the absence of E2, while no changes in TOR protein levels were observed in WT seedlings under the same conditions. TFA content was measured in both *tor-es1* and WT treated with E2 for periods ranging from 2 h to 4 d. As shown in Fig. 2C and Supplementary Fig. S2, TFA levels in *tor-es1* seedlings were significantly reduced after 1 d of E2 treatment and exhibited a 22% decrease after 4 d of treatment compared to nontreated *tor-es* controls. In contrast, no significant difference in TFA content was observed between E2-treated and nontreated WT seedlings at any time point.

### Suppression of TOR activity by Torin 2 significantly reduces both FA and membrane lipid contents

**Figure 3.**
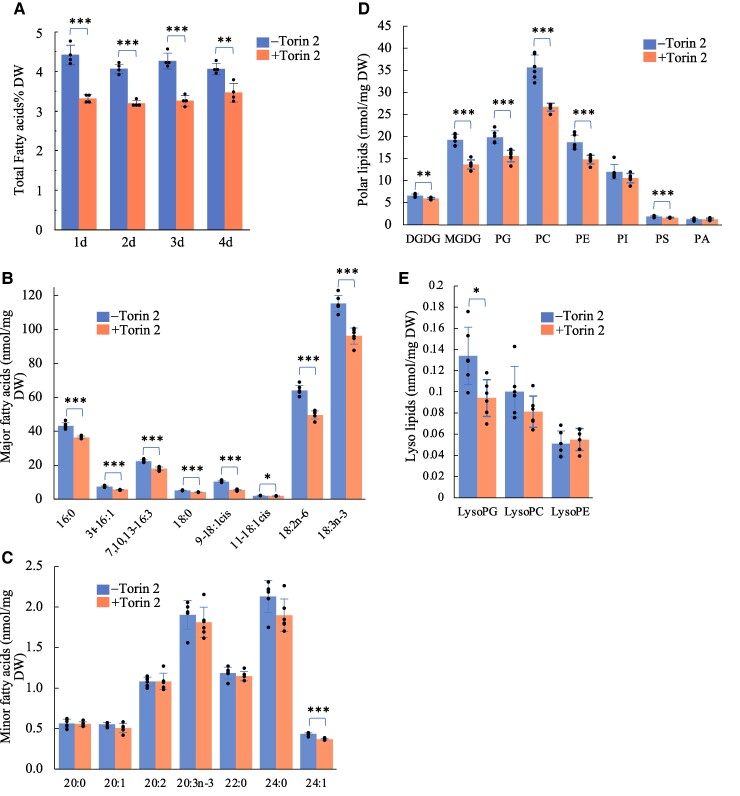
Lipidomics assay shows suppression of TOR activity by Torin 2 reduces the content of FA and membrane lipids. **A)** TFA content in *Arabidopsis* WT seedlings grown in ½ MS liquid medium, supplemented without (−) or with (+)1 *μ*m Torin 2 for the indicated time periods. Lipidomics assays were performed on seedlings treated without or with Torin 2 for 1 d. **B)** Measurement of major FA. **C)** Measurement of minor FA. **D)** Measurement of major polar membrane lipids. **E)** Measurement of lyso lipids. In this figure, bar values represent mean ± Sd (*n* = 4 to 6), with each data point represented by a dot. Asterisks denote statistically significant differences from the non-Torin 2 treatment by Student's *t*-test, *P < 0.05; **P < 0.01; ***P < 0.001. DGDG, digalactosyldiacylglycerol; MGDG, monogalactosyldiacylglycerol; PG, phosphatidylglycerol; PC, phosphatidylcholine; PE, phosphatidylethanolamine; PI, phosphatidylinositol; PS, phosphatidylserine; PA, phosphatidic acid.

**Figure 4.**
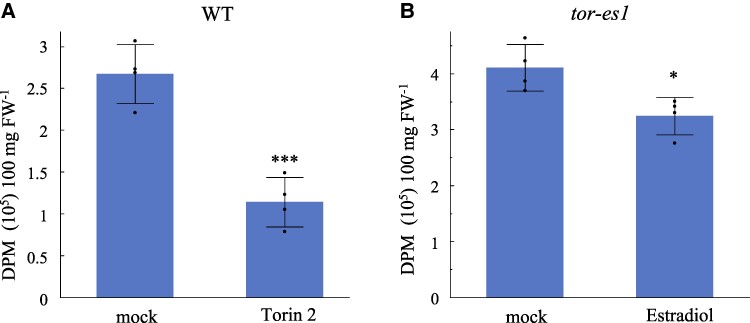
De novo FA synthesis rate is significantly lower in *Arabidopsis* seedlings in which TOR is suppressed. **A)** Four-day-old WT *Arabidopsis* seedlings were transferred into liquid medium (½ MS + 1% sucrose + 0.01% Tween 20) supplemented with either a mock (DMSO) or 1 *μ*m Torin 2 (dissolved in DMSO). After 1 d of treatment, seedlings were labeled with [1-^14^C] acetate for 30 min. Incorporation of ^14^C into newly synthesized FA was quantified by measuring radioactivity in the extracted lipids, expressed as disintegrations per minute (DPM) per 100 mg of fresh weight (FW). The bar graph presents mean values ± Sd (*n* = 4), with each data point represented as a dot. Statistically significant differences between the Torin 2 treatment and the mock controls were determined using Student's *t*-test (*P* < 0.001) and are indicated by asterisks (***). **B)**  *Arabidopsis tor-es1* seedlings were treated with (mock) or without 1 *μ*m estradiol in liquid medium for 1 d before [1-^14^C] acetate labeling. The bar graph presents mean values ± Sd (*n* = 4), with each data point represented as a dot. Statistically significant differences between the estradiol treatment and the mock controls were determined using Student's *t*-test (*P* < 0.05) and are indicated by asterisk (*).

To assess whether the suppression of TOR activity by chemical inhibition produces effects similar to those observed with the inducible TOR silencing, *Arabidopsis* WT seedlings were treated with Torin 2, a specific ATP-competitive inhibitor of TOR (Liu et al. 2013; Yuan et al. 2020). In agreement with previous studies (Yuan et al. 2020), *Arabidopsis* seedlings demonstrated high sensitivity to Torin 2 treatment at nanomolar concentrations (Supplementary Fig. S3). Torin 2-treated seedlings showed a significant reduction in TFA levels, with an ∼25% decrease compared with mock-treated seedlings after 1 d, and this reduction persisted over the 4-d period (Fig. 3A; Supplementary Fig. S4). These effects closely mirrored those observed with inducible gene silencing of TOR. To further explore the role of TOR in regulating lipid metabolism, we performed lipidomic analysis on WT seedling treated with Torin 2 for 1 d, comparing the results with mock controls. Most detectable FA were significantly reduced in Torin 2-treated seedlings (Fig. 3, B and C). For instance, the major FA such as 18:3 and 16:3 exhibited reductions of 17% and 19%, respectively, after 1 d of Torin 2 treatment. A similar trend was observed for the membrane lipids, with the majority showing significant reductions following Torin 2 treatment, ranging from 10% to 30% (Fig. 3, D and E). The observed reductions in various FA and polar lipids upon Torin 2 treatment suggest that de novo FA synthesis is impaired by TOR inhibition. To directly test this hypothesis, we conducted a lipid synthesis rate assay using [^14^C] acetate labeling in Torin 2-treated WT seedlings or estradiol-treated *tor-es1*. The results demonstrated significantly lower incorporation of ^14^C into lipids in either Torin2-treated seedlings or estradiol-treated *tor-es1* compared with the respective mock treatment during a 30-min labeling period, confirming that TOR inhibition reduces de novo FA synthesis (Fig. 4).

### Torin 2 treatment of *B. napus* suspension cell culture resulted in significant decreases in the accumulation of both TFA and TAG

**Figure 5.**
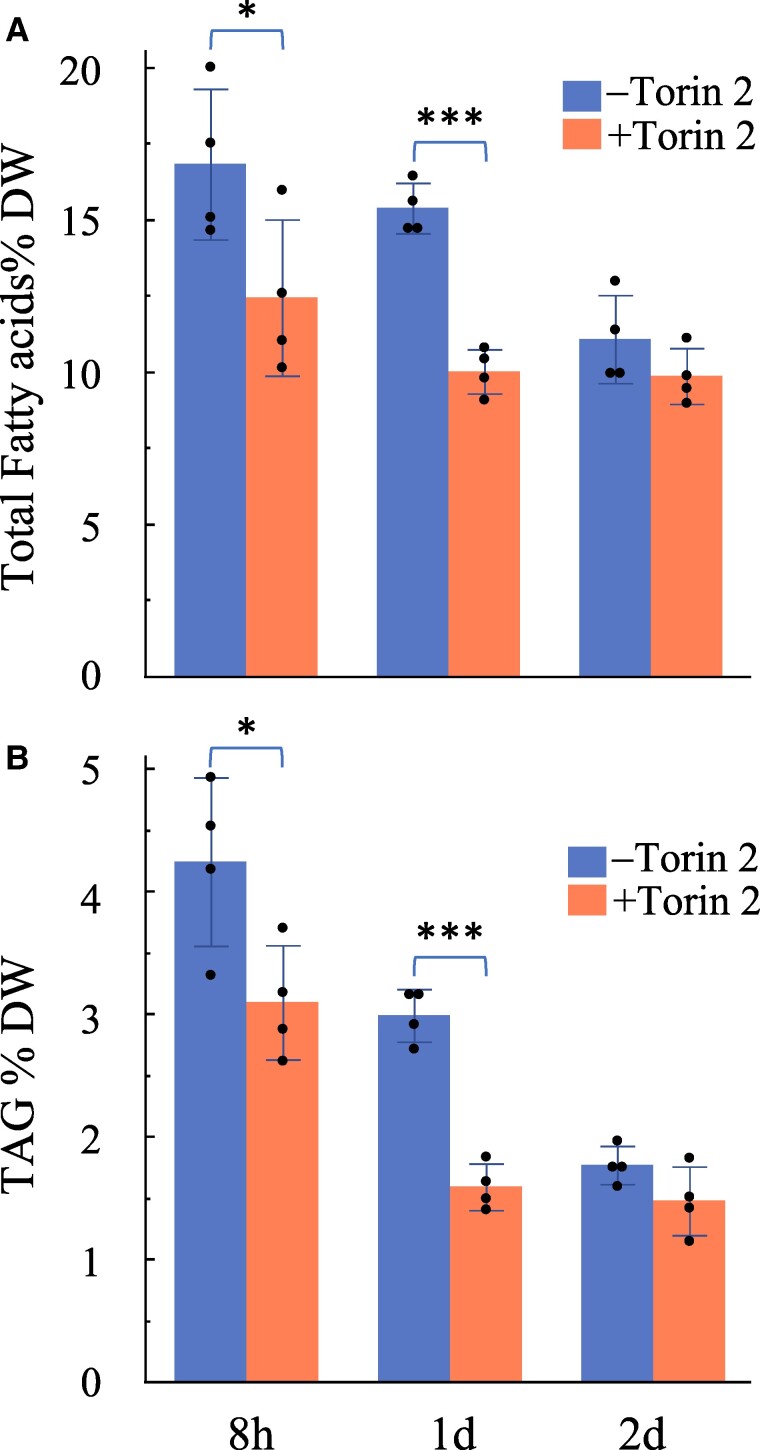
Suppression of TOR activity by Torin 2 in a *B. napus* suspension cell culture results in significant reduction in the accumulation of both TFA and TAG. **A)** TFA content in *B. napus* microspore-derived suspension cells cultured in medium supplemented without (−) or with (+) 1 *μ*m Torin 2 for the indicated time periods. **B)** TAG content in the *B. napus* suspension cells in **A)**. In this figure, bar values represent mean ± Sd (*n* = 4), with each data point represented by a dot. Asterisks denote statistically significant differences from the non-Torin 2 treatment controls (Student's *t*-test, *P < 0.05; ***P < 0.001).

As previously shown, TOR promotes the accumulation of FA and membrane lipids in vegetative tissues, such as leaves and seedlings. To investigate the regulatory role of TOR in storage lipid triacylglycerol (TAG) metabolism, we utilized a well-established model system: *B. napus* cv. Jet Neuf suspension cells. Unlike vegetative tissues, which contain negligible amounts of TAG under normal growth conditions, *B. napus* suspension cells, derived from embryogenic microspores, accumulate ∼4% of TAG per dry weight, accounting for nearly half of their total lipid content (Taylor et al. 1990; Weselake et al. 1993; Shi et al. 2008). Torin 2 treatment began immediately after the suspension cells were transferred to fresh medium. Total lipids were extracted from *B. napus* suspension cells treated without or with 1 *µ*m Torin 2 for 8 h, 1 d, or 2 d. Both TFA and TAG were subsequently analyzed. As shown in Fig. 5, Torin 2 treatment significantly reduced the accumulation of both TFA and TAG in suspension cells compared with untreated controls, with notable reductions observed after 8 h or 1 d of treatment. Specifically, after 1 d of Torin 2 treatment, TFA decreased by 35%, while TAG accumulation dropped by 47% (Fig. 5). However, after 2 d of treatment, no significant differences in TFA or TAG levels were observed between Torin 2-treated and untreated suspension cells, likely due to sucrose depletion in the culture medium, which may have contributed to the cessation of lipid accumulation.

### RNA-seq analysis of WT seedlings treated with Torin 2

**Figure 6.**
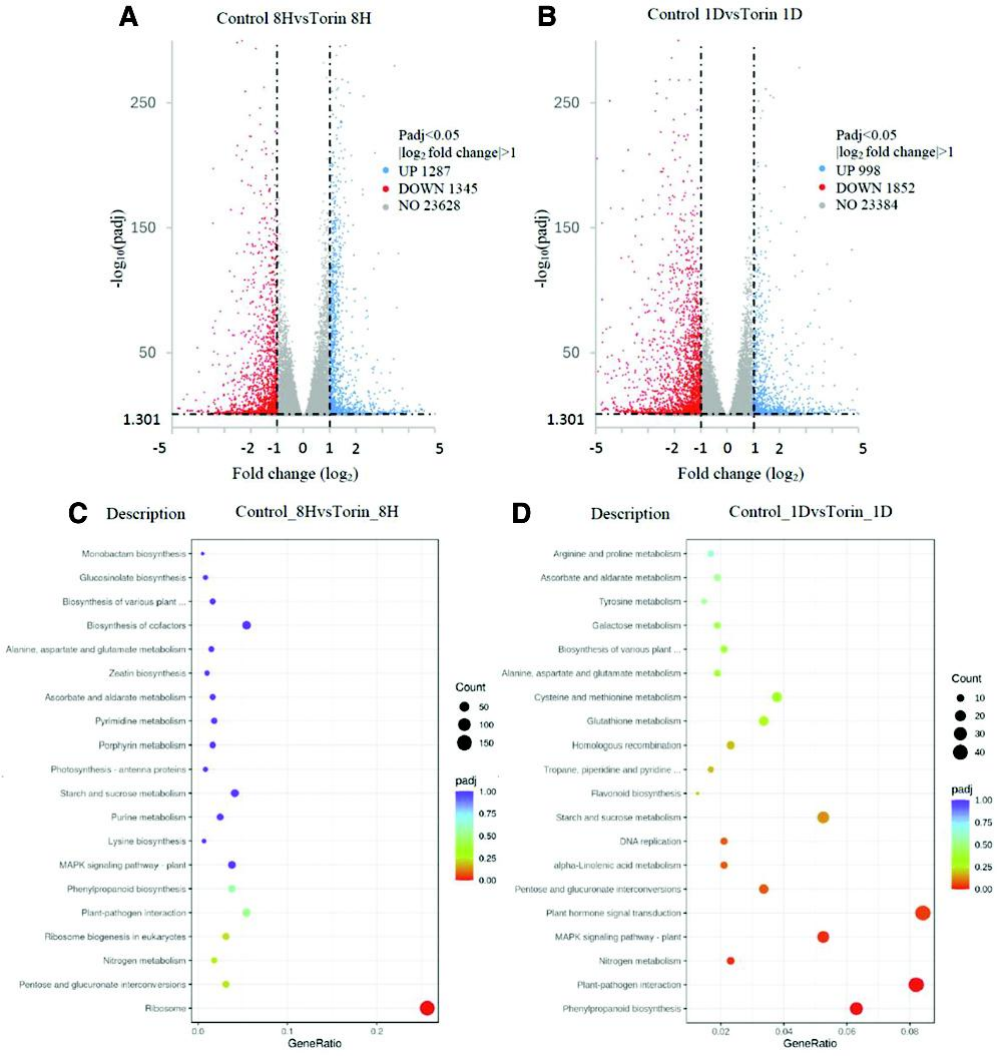
RNA-seq analysis of WT seedlings treated with Torin 2 volcano plots show DEGs for mock controls compared with Torin 2 treatments for 8 h **A)** or 1 d **B)**. Bubble plot KEGG pathway analysis of DEGs for mock controls compared with Torin 2 treatments for 8 h **C)** or 1 d **D)**. “Count” indicates the number of DEGs enriched in a pathway. “GeneRatio” indicates the ratio of count of enriched DEGs to count of pathway genes. “p.adj” indicates the adjusted *P*-value.

To gain deeper insights into how TOR regulates plant lipid metabolism, we conducted an RNA-seq analysis on *Arabidopsis* WT seedlings treated with 1 *µ*m Torin 2 for either 8 h or 1 d. After 8 h of treatment, a comparison between mock controls with Torin 2-treated seedlings revealed that 2,632 genes were differentially expressed, with 1,287 genes upregulated and 1,345 genes downregulated (Fig. 6A). Following 1 d of Torin 2 treatment, 998 genes were upregulated, and 1,852 genes were downregulated (Fig. 6B). The complete RNA-seq data for all *Arabidopsis* genes are provided in Supplementary File S2.

Kyoto Encyclopedia of Genes and Genomes (KEGG) pathways and gene ontology (GO) enrichment analysis for differentially expressed genes (DEGs) following 8 h of Torin 2 treatment showed TOR primarily regulates protein synthesis. Upregulated genes were enriched for those encoding the large and small ribosomal subunits, rRNA, ribosome biogenesis, and cytoplasmic translation. Additionally, genes involved in the pentose phosphate pathway, nitrogen metabolism, phenylpropanoid biosynthesis, amino acid biosynthesis, nucleotide biosynthesis, and starch and sucrose metabolism were also upregulated. In contrast, downregulated genes included those related to cellular responses to hypoxia, wounding, bacterial and fungal defense, oxidative stress, and hormone responses to salicylic acid, abscisic acid, jasmonic acid, as well as genes involved in leaf senescence and osmotic stress (Fig. 6C; Supplementary Fig. S5A).

After 1 d of Torin 2 treatment, genes upregulated were enriched in pathways related to cell wall biosynthesis, including phenylpropanoid biosynthesis and cell wall protein biosynthesis, the MAP kinase signaling pathway, plant hormone signal transduction, lipid synthesis, and DNA replication. Meanwhile, genes involved in stress responses continued to be downregulated (Fig. 6D; Supplementary Fig. S5B).

The DEGs involved in lipid metabolism identified during Torin 2 treatment are summarized in Table 1 (the complete list of lipid metabolism genes analyzed for differential expression under Torin 2 treatment is available in Supplementary File S3). In alignment with the observed reduction in TFA and lipid contents in Torin 2-treated seedlings, several genes encoding enzymes involved in de novo FA synthesis, such as plastidic acetyl-CoA carboxylase 2 (ACC2), stearoyl-ACP desaturase (FAB2), ketoacyl-ACP Synthase I (KASI), and acyl carrier protein (ACP), were significantly downregulated by Torin 2. In contrast, the expression of genes encoding lipases involved in lipid turnover, such as monoacylglycerol lipases (MAGL), TAG lipases (TAGL), and phospholipase A2 (PLA), was significantly upregulated upon Torin 2 treatment. Additionally, an increase in transcripts associated with epidermal lipid and glycolipid syntheses was noted, potentially indicating a stress response to Torin 2. To validate RNA-seq data, reverse transcription quantitative PCR (RT-qPCR) analysis was conducted for the selected DEGs, including *MAGL* (AT5G18640), *TAGL* (AT5G67050), *ACP* (AT4G25050), *FAB2*(AT1G43800), and *KAS I* (AT5G46290). As shown in Supplementary Fig. S6, RT-qPCR results were consistent with the RNA-seq data: expressions of *MAGL* and *TAGL* were significantly upregulated, while expressions of *ACP*, *FAB2*, and KAS *I* were significantly repressed by Torin 2 treatment.

**Table 1.** A list of genes involved in FA and lipid metabolism are differentially regulated by TOR

Subsystem in lipid metabolism	Abbreviation	Name	Ath Gene	8 h (H)	1 d (D)
Acetyl-CoA carboxylase complex	ACC2 (plastid)	Homomeric acetyl-CoA carboxylase	AT1G36180	n/a	Down in Torin 1D
Acylglycerol lipase	MAGL	Monoacylglycerol lipase	AT2G39420	Up in Torin 8H	n/a
Acylglycerol lipase	MAGL; LPLA	Monoacylglycerol lipase; lipoprotein lipase	AT5G14980	Up in Torin 8H	n/a
Acylglycerol lipase	TAGL	Triacylglycerol lipase	AT5G14180	n/a	Up in Torin 1D
Acylglycerol lipase	TAGL	Triacylglycerol lipase	AT5G14930	n/a	Up in Torin 1D
Acylglycerol lipase	TAGL	Triacylglycerol lipase	AT5G18640	Up in Torin 8H	n/a
Acylglycerol lipase	TAGL	Triacylglycerol lipase	AT5G42930	n/a	Up in Torin 1D
Acylglycerol lipase	TAGL	Triacylglycerol lipase	AT5G67050	Up in Torin 8H	n/a
De novo FA synthesis	AAE15/16	Acyl-ACP synthetase	AT3G23790	Up in Torin 8H	Up in Torin 1D
De novo FA synthesis	ACP	Acyl carrier protein	AT4G25050	Down in Torin 8H	n/a
De novo FA synthesis	FAB2/SAD1-6	Stearoyl-ACP desaturase	AT1G43800	Down in Torin 8H	n/a
De novo FA synthesis	KAS I	Ketoacyl-ACP Synthase I	AT5G46290	Down in Torin 8H	n/a
Epidermal lipid synthesis	KCR	Ketoacyl-CoA reductase	AT1G24470	n/a	Up in Torin 1D
Epidermal lipid synthesis	KCS	Ketoacyl-CoA synthase	AT1G04220	Up in Torin 8H	Up in Torin 1D
Epidermal lipid synthesis	KCS	Ketoacyl-CoA synthase	AT4G34510	Up in Torin 8H	n/a
Glycerolipid synthesis	GPAT	Glycerol-3-phosphate acyltransferase 1 (GPAT1)	AT3G11430	Up in Torin 8H	Up in Torin 1D
Glycerolipid synthesis	GPAT	Glycerol-3-phosphate acyltransferase 1 (GPAT1)	AT5G06090	Up in Torin 8H	Up in Torin 1D
Glycerolipid synthesis	LPAAT	Lysophosphatidic acid acyltransferase	AT1G75020	n/a	Up in Torin 1D
Glycerolipid synthesis	PDCT	Phosphatidylcholine:diacylglycerol cholinephosphotransferase	AT3G15820	n/a	Up in Torin 1D
Glycerolipid synthesis	PLA	Phospholipase A2	AT4G19860	Up in Torin 8H	Up in Torin 1D
Glycerolipid synthesis	PLA	Phospholipase A2	AT4G29070	Up in Torin 8H	Up in Torin 1D
Glycerolipid synthesis	PP	Phosphate phosphatase	AT2G01180	n/a	Up in Torin 1D

n/a, not available.

## Discussion

TOR kinase plays a crucial role in regulating cell growth. Generally, TOR is activated to promote cell growth (expansion and proliferation) when environmental conditions and cellular signals are favorable, while it is suppressed to inhibit cell growth and induce cell death (via autophagy) under stress conditions (Liu and Bassham 2010). These functions are involved in the synthesis and remodeling of lipids, ensuring proper membrane structure and function and for energy storage. Previous studies demonstrated that prolonged inhibition of TOR by gene silencing leads to accumulation of the storage lipid TAG in plant vegetative tissues (Caldana et al. 2013).

To understand the primary regulatory role of TOR in lipid metabolism, especially how TOR regulates membrane lipids, and storage lipid TAG in tissues that normally produce TAG, we tracked the changes of TFA, membrane lipids, and TAG in the several plant systems in which the TOR protein levels or activities were disrupted for brief time periods. Several independent lines of evidence are consistent with a primary function of TOR as promoting FA and lipid synthesis and accumulation in plants. These include the following: (i) transient expression of TOR significantly increases the TFA content in *N. benthamiana* leaves. (ii) Suppression of TOR, either by inducible gene silencing or treatment with a TOR-specific inhibitor, significantly reduces most FA and membrane lipids in *Arabidopsis* seedlings. (iii) Suppression of TOR by treatment with a TOR inhibitor leads to significant reductions in both TFA and TAG contents in *B. napus* embryogenic suspension cells. These data are seemingly at odds with a previous study in which suppression of TOR led to accumulation of TAG in vegetative tissues. However, the data presented here are based on data at earlier timepoints and from 3 different experimental systems, which show a consistent pattern of TOR stimulating fatty and lipid accumulation. We note that it makes biological sense for TOR to promote FA synthesis because FA are key substrates for de novo membrane synthesis which is required for cell growth. TAG accumulation in vegetative tissue is a common response to cellular stresses such as biotic or abiotic stress and senescence. For example, fungus infections result in accumulation of PUFA-abundant TAGs that subsequently serve as substrates for producing defense compounds (Shimada and Hara-Nishimura 2015); During cold acclimation, TAGs containing LC-PUFA also accumulate during membrane remodeling (Degenkolbe et al. 2012). Therefore, we recognize the possibility that former report of TAG accumulation may have been the result of an indirect stress response incurred upon prolonged suppression of TOR as previously reported in algae (Imamura et al. 2015).

Regarding the mechanism by which TOR promotes lipid synthesis, in this study, we did not observe a significant decrease in TFA content in Torin 2-treated WT or estradiol-treated *tor-es1* until 1 d of treatment. Similarly, it took 8 h to observe decreased TFA and TAG in *B. napus* suspension cells treated with Torin 2. These results suggest crucial enzymes involved in FA synthesis or turnover are unlikely to be the direct protein substrates of TOR because ribosomal protein S6 kinase 1 (S6K1), a well-defined substrate of TOR is phosphorylated within minutes of TOR activation by sucrose, resulting in a dramatic increase in its activity (Van Leene et al. 2019). Consistent with our hypothesis, only one enzyme involved in the synthesis of diacylglycerol, a key TAG intermediate, namely PHOSPHATIDIC ACID PHOSPHOHYDROLASE 2, was phosphorylated within 40 min of TOR activation in a large-scale phosphoproteomics study (Van Leene et al. 2022). The delay we observed between the modulation of TOR activity and changes in FA and lipid contents are more consistent with it playing a role in the regulation of gene expression and de novo protein synthesis. Consistent with this, our RNA-seq data from WT seedlings treated with Torin 2 clearly show that TOR regulation shifts from dominant protein synthesis in the early stage, to cell wall synthesis, lipid synthesis, DNA replication, and secondary metabolism at later time points. In our lipidomic assays, we also observed that the contents of most FA types and membrane lipid species decreased to a similar extent in WT seedlings treated with Torin 2 for 1 d when compared with a mock treatment, suggesting that de novo FA synthesis and/or turnover is affected by TOR inhibition. TOR promoting de novo FA synthesis is supported by lipid synthesis rate assay using [^14^C] acetate labeling in which the synthesis rate of nascent FA is significantly reduced by TOR inhibition. Additionally, the analysis of genes involved in plant lipid metabolism that is differentially expressed in Torin 2-treated seedling also shows that TOR inhibition leads to downregulation of enzymes in de novo FA synthesis and upregulation of enzymes involved in lipid turnover, which indicates a switch in the balance from lipid synthesis to lipid degradation that explains the observed decrease in FA and lipid accumulation levels. WRINKLED1 (WRI1) is a seed-specific master transcription factor for oil synthesis (Focks and Benning 1998; Cernac and Benning 2004). In contrast, TOR is more widely expressed across different plant tissues. In our RNA-seq analysis, WRI1 was not identified as a differentially expressed gene, suggesting that TOR-mediated FA and lipid synthesis may function in parallel to the WRI1 regulatory pathway.

**Figure 7.**
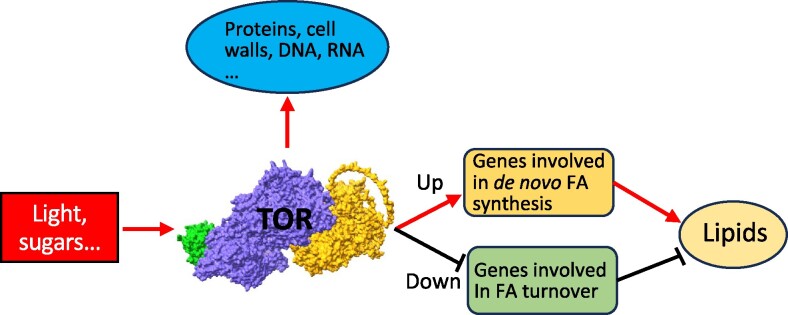
TOR kinase is a positive regulator of plant FA (FA) and lipid synthesis. Plant TOR is activated by light and sugars. Once activated, TOR positively regulates FA and lipid synthesis by upregulating genes involved in de novo FA synthesis and downregulating genes associated with lipid turnover. Additionally, TOR promotes the biosynthesis of proteins, cell walls, DNA, RNA, and the other essential cellular components. TOR complex shown in the figure is comprised of LST8-1 (lethal with SEC13 protein 8-1, left), TOR (middle), and RAPTOR1B (regulatory-associated protein of TOR 1B, right), and its 3D structure model is predicated by AlphaFold 3.

Taken together, like the well-defined role of mTORC1 in promoting lipid synthesis and storage in mammals, we propose (i) that plant TOR likewise promotes lipid synthesis and accumulation in plant tissues and (ii) that it achieves this by upregulating the expression of genes involved in de novo FA synthesis while downregulating genes involved in lipid turnover (Fig. 7).

## Materials and methods

### Plant material and culture conditions

*A. thaliana* Columbia-0 (WT), transgenic lines with estradiol-inducible RNA interference (RNAi) against TOR (tor-es1 and tor-es2; ABRC Stock Numbers: CS69829 and CS69830), *N. benthamiana*, and microspore-derived cell suspension culture of *B. napus* were used in this study. For *Arabidopsis* seed germination, seeds were surface sterilized with 70% ethanol followed by 30% bleach (Clorox) containing 0.01% Tween 20 for 15 min. Seeds were rinsed 5 times with sterile water before being planted on half-strength MS medium supplemented with 1% sucrose and solidified by adding agar to 0.7% (W/V). After stratification at 4 °C in the dark for 3 d, seeds were germinated and cultured in a Percival plant tissue culture chamber under a 16-h light/8-h dark cycle at 24 °C/22 °C, with a photosynthetic photon flux density of 250 *μ*m m^−2^ s^−1^ and 75% relative humidity.

### Genetic construct and *N. benthamiana* leaf agroinfiltration

The coding sequence of *Arabidopsis TOR* was amplified with forward primer (5′CGGGGGACGAGCTCGATGTCTACCTCGTCGCAATCT3′) and reverse primer (5′CGCCGCCGCCTCTAGACCAGAAAGGGCACCACCCA3′) and cloned into pCHF3-hGFP between *Knp*I and *Xba*I by In-fusion cloning (Takara). The resulting TOR/pCHF3-hGFP construct was then transformed into Agrobacterium GV3101. WRI1-GFP construct was described previously in Zhai et al. (2023) Agroinfiltration in *N. benthamiana* was performed as described by Gehl et al. (2009).

### Arabidopsis seedling estradiol or Torin 2 treatment

Three-day-old WT or *tor-es1* seedlings were germinated and grown on solid ½ MS (containing 1% W/V of sucrose) plates and were transferred to liquid ½ MS (1% sucrose) medium in multiple-well cell culture plate. After 1 d of acclimation, estradiol or Torin 2 treatments were initiated by adding estradiol or Torin 2 (solubilized in DMSO) stock solution to the liquid medium to a final concentration of 1 *μ*m. An equal volume of DMSO was added into liquid medium as the mock treatment (negative control). After specified periods, seedings were harvested and frozen in liquid nitrogen and stored at −80 °C until analysis.

### Generation of TOR antibody

The coding sequence corresponding to the last 200 amino acids of TOR's C-terminus (aa2282 to 2481), referred to as TORC, was cloned into *E. coli* expression vector pCold trigger factor (TF; catalog no. 3365, Takara Bio). Recombinant TF-TORC fusion protein was expressed in *E. coli* BL21 (DE3) and purified as described by Nallamsetty and Waugh (2007). The identity of TORC was confirmed by digesting TF-TORC with human rhinovirus 3c protease, which recognizes a sequence between TF and TORC. A customized polyclonal antibody against TOR was produced by GenScript (Piscataway, NJ, USA) using purified TF-TORC protein as the antigen.

### IP of native TOR from Arabidopsis

For native TOR IP from *Arabidopsis*, 1 g of 7-d-old WT seedlings grown on ½ MS plate were harvested and ground into a fine powder using a mortar and pestle with the addition of liquid nitrogen. The resulting tissue powder was transferred to a precooled 15 mL conical centrifuge tube (Falcon) and mixed with 2 mL of protein extraction buffer (50 mm Tris-HCl [pH8.0], 150 mm NaCl, 10% glycerol, 1% [V/V] NP-40, 0.15% [W/V] BSA, protease inhibitor cocktail [cOmplete, Minim EDTA-free, Roche], PhosSTOP [Roche]). Total proteins were extracted for 15 min at 4 °C with rotation, followed by 2 sequential clarifications by centrifugations at 34,000 × *g*. The resulting supernatant was concentrated using a 100 kDa cutoff Amicon Ultra Filter (Merck Millipore, Billerica, MA, USA) via centrifugation at 3,500 × *g*. A 250-*μ*L aliquot of concentrated total protein was mixed with 5 *μ*g of TOR antibody and incubated at 4 °C for 2 h with gentle agitation to permit antibody–antigen complex formation. The complex was then captured by incubating with Dynabeads Protein G (Thermo Fisher Scientific), followed by magnetic separation according to the manufacturer's instruction. The captured complex was washed 3 times in IP buffer (25 mm Tris-HCl [pH 8.0], 150 mm NaCl, and 0.15% [V/V] NP-40) before elution with glycine buffer (0.2 m and pH 2.6). The eluate was neutralized by the addition of Tri-HCl (pH 8.0).

### Immunoblotting

Immunoblotting is performed as previously described in (Zhai et al. 2023). Briefly, 20 mg of freshly harvested seedlings or leaf tissue was ground in liquid nitrogen and mixed with 80 *µ*L of protein extraction buffer (8 m urea, 2% SDS, 0.1 m DTT, 20% glycerol, 0.1 m Tris-HCl, pH [6.8], and 0.004% Bromophenol Blue). After incubation at 80 °C for 5 min, crude protein extract samples were clarified by centrifugation at 17,000 × *g*, and proteins in 10 *μ*L of the resulting supernatant were separated by SDS-PAGE. Proteins were transferred to PVDF membrane, and the membrane blocked by incubation in a 5% (W/V) milk. For primary antibody probing, anti-TOR was used at a 1:3,000 dilution, anti-GFP 1:2,000 (catalog no. A6455, Invitrogen). After probing with horseradish peroxidase-conjugated secondary antibodies (1:10,000 dilution; catalog no. AS09 602, Agrisera), targeted proteins were visualized using SuperSignal West Femto Maximum Sensitivity Substrate (catalog no. 34095, ThermoFisher). Immunoblot signals were detected and digitalized with Amersham ImageQuant 800 (Cytiva).

### RNA isolation and RNA-seq and RT-qPCR

Total RNA (*n* = 6) was isolated from *Arabidopsis* seedlings treated with mock or Torin 2 for 8 h or 1 d using the RNeasy Plant Mini Kit (Qiagen). RNA samples were stored at −80 °C and shipped on dry ice to Novogene for sequencing. RT-qPCR was conducted according to the method described in Zhai et al. (2023).

### Differential expression analysis, KEGG pathway, and GO term enrichment analysis

Differential expression between control and Torin treatment was performed using the DEseq2 package (Love et al. 2014). Significant differential expressed genes were defined for an adjusted *P*-value ≤5% (Benjamini–Hochberg adjustment for multiple testing) and a more than 2-fold change in gene expression between conditions. KEGG pathway enrichment analysis using KEGG pathway database and GO term enrichment analysis using the Database for Annotation, Visualization and Integrated Discovery (Huang et al. 2009; Sherman et al. 2022).

### Lipid extraction and TFA and TAG analyses

Lipid extraction and TFA and TAG analyses are conducted as previously described in the study by Cai et al. (2022). Total lipids were isolated from 100 mg of fresh leaf tissue (*N. benthamiana*) or 10 mg of freeze-dried tissue (*Arabidopsis* seedlings and *Brassica* suspension cells) with 700 *μ*L of methanol:chloroform:formic acid (2:1:0.1, V/V/V). The mixture was vigorously vortexed for 1 h and left to stand at room temperature overnight. After the addition of 1 m KCl and 0.2 m H_3_PO_4_, samples were shaken using a vortex mixer and subjected to centrifugation at 2,000 × *g* for 10 min. The lower phase containing the lipids was collected for further analysis. For TFA analysis, 10 *µ*L of total lipid extract was trans-methylated to FA methyl esters (FAMEs) by incubation in 1 mL of boron trichloride-methanol (V/V) at 80 to 85 °C for 90 min. For TAG quantification, total lipid extracts were separated by thin-layer chromatography using a hexane/diethyl ether/acetic acid (70:30:1, v/v/v) solvent system on Silica Gel 60 plates (EMD Millipore). Lipids were visualized by spraying with 0.05% primulin (in 80% acetone). TAG fractions were identified under UV light by comparing its mobility with that of TAG standards, excised from the plate, and trans-methylated to FA methyl esters by incubation in 1 mL of boron trichloride-methanol. To enable quantification, 5 *μ*g of C17:0 was added as an internal standard prior to transmethylation. FAMES were extracted into hexane, dried under a stream of nitrogen, and dissolved in 100 *μ*L of hexane. FAMES were analyzed by gas chromatography-mass spectrometry using an Agilent Technologies 7890A GC System equipped with a 5975C mass selective detector and a 60-m × 0.25-mm inside diameter DB23 column.

### Lipid extraction for lipidomics assays

One volume of isopropanol containing 0.01% butylated hydroxytoluene was added to leaf tissues and incubated at 75 °C for 15 min to inactivate lipolytic enzymes. After cooling to room temperature, 3 volumes of a chloroform:methanol:water mixture (30:41.5:3.5, V/V/V) were added and shaken at 100 rpm for 24 h at room temperature. The extracts were transferred to a new vial, and the tissues were re-extracted a second time with 3 volumes of chloroform:methanol:water (30:66.5:3.5, V/V/V), immediately after the first extraction, with shaking for 48 h. The 2 extracts were combined, dried under nitrogen, and weighed. The extracted tissues were dried overnight at 105 °C and weighed. The extracts were redissolved in 1 mL of chloroform, transferred to clear glass vials, and evaporated completely under nitrogen before being shipped on dry ice to the Kansas Lipidomics Research Center for lipidomics assays.

### De novo FA synthesis assay

The de novo FA synthesis assay with in vivo [1-^14^C] acetate labeling was conducted following the method described by Zhai et al. (2017). Arabidopsis seedlings (0.1 g fresh weight) were incubated in ½ MS containing 0.01% (w/v) Tween 20 as wetting agent, under constant illumination (180 *µ*mol m^−2^ s^−1^) at 25 °C. Labeling was initiated by adding 0.02 mCi of [1-^14^C] acetate acid sodium (1 mCi/mL, American Radiolabeled Chemicals) to the medium. After 30 min of incubation, the labeling was terminated by removing the medium, and the seedlings were washed 3 times with deionized water. Total lipids were then extracted and separated as described in lipid analysis section. Radioactivity associated with extracted lipids was measured using a Tri-carb liquid scintillation counter (Perkin-Elmer).

## Accession numbers

Sequence data from this article can be found in the GenBank/EMBL data libraries under accession numbers AT1G50030 (TOR) and AT3G54320 (WRI1).

## Supplementary Material

kiae639_Supplementary_Data

## Data Availability

The data underlying this article will be shared on reasonable request to the corresponding author.

## Supplementary data

The following materials are available in the online version of this article.

**Supplementary Figure S1.** Generating TOR antibody and testing its specificity.

**Supplementary Figure S2.** TFA analysis in *Arabidopsis* WT and *tor-es1* seedlings grown with estradiol (E2).

**Supplementary Figure S3.** Phenotypes of WT seedlings grown with Torin 2.

**Supplementary Figure S4.** TFA contents in *Arabidopsis* WT seedlings treated with Torin 2.

**Supplementary Figure S5.** GO enrichment analysis.

**Supplementary Figure S6.** RT-qPCR analysis of the selected lipid metabolism genes identified in the differential expression analysis during Torin 2 treatment.

**Supplementary File S1.** Protein sequencing results for immunoprecipitated TOR.

**Supplementary File S2.** List of all the genes in RNA-seq assay.

**Supplementary File S3.** Differential expression analysis among lipid metabolism genes for torin treatment.

## Dive Curated Terms

The following phenotypic, genotypic, and functional terms are of significance to the work described in this paper:

ACC2 Gramene: AT1G36180
ACC2 Araport: AT1G36180

FAB2 Gramene: AT2G43710
FAB2 Araport: AT2G43710
