# Supplementary material for: The target of rapamycin kinase is a positive regulator of plant fatty acid and lipid synthesis
Source: Plant Physiol. 2024 Dec 2;197(2):kiae639. doi: 10.1093/plphys/kiae639 (PMC11809584; doi:10.1093/plphys/kiae639)
Supplement: kiae639_Supplementary_Data [file kiae639_supplementary_data.zip › Supplementary Data.pdf]

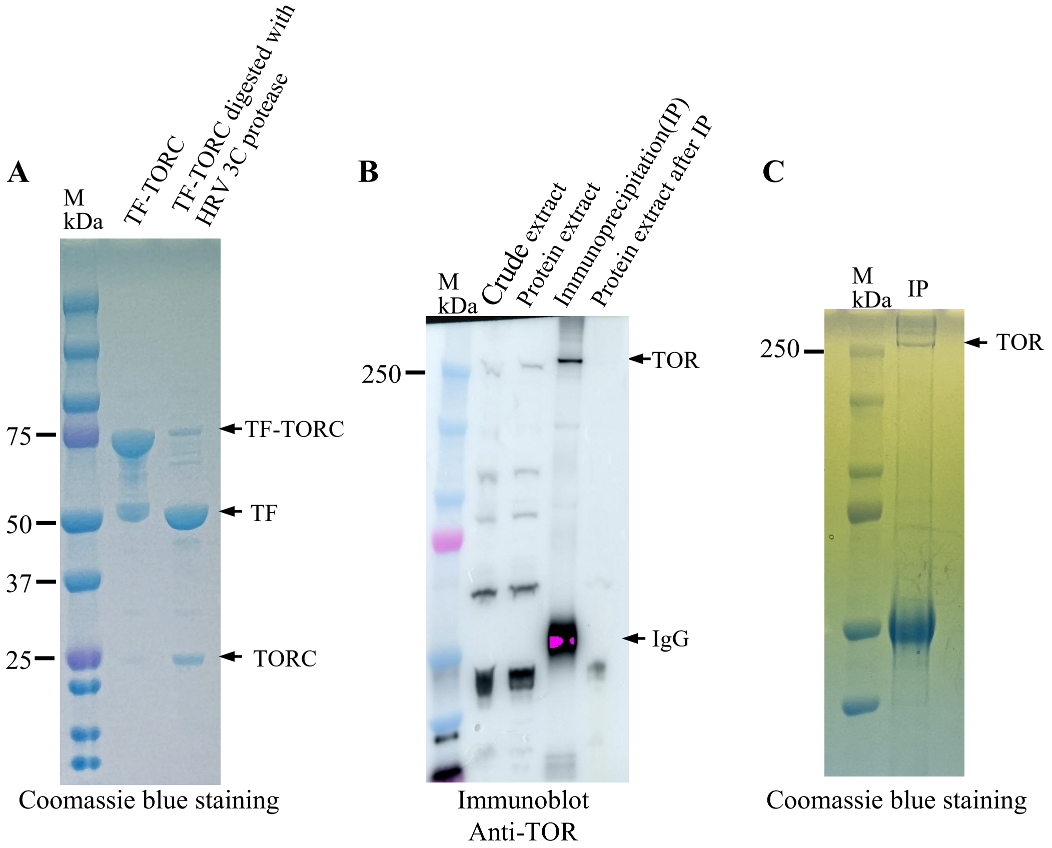

Supplementary Figure S1. Generating TOR antibody and testing its specificity

A) A partial TOR polypeptide (aa2282-2481, the last 200 amino acid residues from the TOR's C-terminal, referred to as TORC) was produced and purified as a trigger factor (TF) fusion protein (TF-TORC) from *E. coli*, and used as an antigen to generate the TOR antibody. The presence of TORC was confirmed by digesting TF-TORC with Human Rhinovirus (HRV) 3C Protease, which recognizes a cleavage site sequence between TF and TORC. 2 μg of TF-TORC or TORC digested by HRV 3C was loaded and run using SDS-PAGE (SurePAGE™, Bis-Tris, and stained with Coomassie Blue (ProBlue Safe Stain, Giottobiotech). **B-C)** To test the specificity of the TOR antibody, immunoprecipitation (IP) was performed using protein extracts from one-week-old wild-type Arabidopsis seedlings, incubated with the TOR antibody. Following a 2h incubation at 4°C, Dynabeads™ Protein G was added to recover the TOR-antibody complex using the manufacturer's protocol (Thermo Fisher Scientific). **B)** Immunoblot probed with the TOR antibody shows a protein larger than 250 kDa was enriched after IP. **C)** The enriched protein, harvested from the SDS-PAGE gel was stained with Coomassie Blue, and subjected to protein sequencing by mass spectrometry to confirm its identity as TOR (S. File 1).

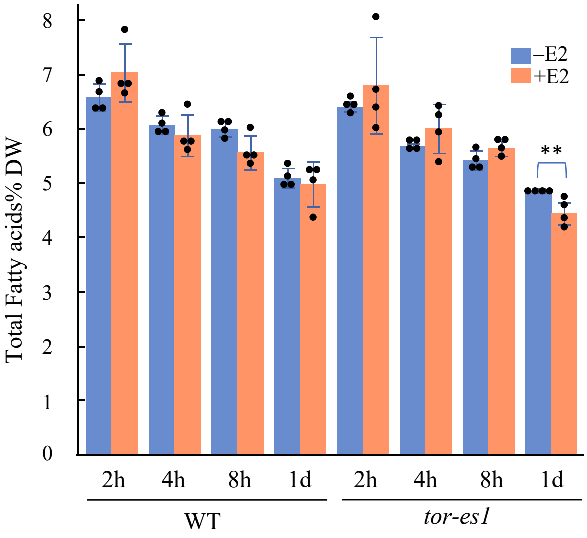

Supplementary Figure S2. TFAs analysis in Arabidopsis WT and *tor-es1* seedlings grown with estradiol (E2)

TFAs content in Arabidopsis WT and *tor-es1* seedlings grown in liquid medium (½ MS + 1% sucrose) supplemented without (−) or with (+) 1 µM of estradiol (E2) for 2 hours (h), 4 hours, 8 hours, or 1 day. Bar values represent mean ± SD (n = 4), with each data point represented by a dot. Asterisks denote statistically significant differences from non-E2 treated negative controls (Student's t-test, **, $P < 0.01$).

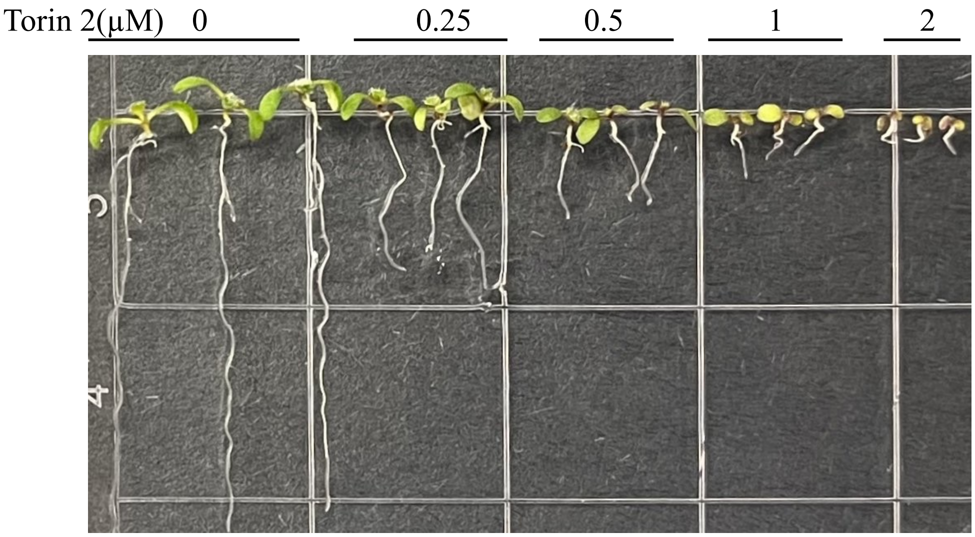

Supplementary Figure S3. Phenotypes of WT seedlings grown with Torin 2

Representative phenotypes of WT Arabidopsis seedlings germinated and grown on ½ MS (1% sucrose) supplemented with various concentrations of Torin 2 (0, 0.25, 0.5, 1, or 2 µM) at 24°C for 7 days. Unit grid length = 13.5 mm.

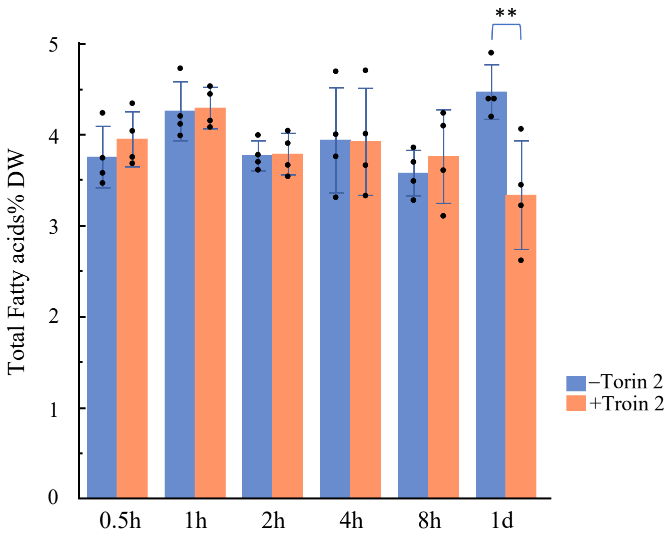

Supplementary Figure S4. TFA contents in Arabidopsis WT seedlings treated with Torin 2

TFAs content in Arabidopsis WT seedlings grown in liquid medium (½ MS + 1% sucrose) supplemented without (−) or with (+) 1 μM Torin 2 for the indicated times. Bar values represent mean ± SD (n = 4), with each data point represented by a dot. Asterisks denote statistically significant differences between the Torin 2 treatment and negative controls (Student's t-test, **, $P < 0.01$).

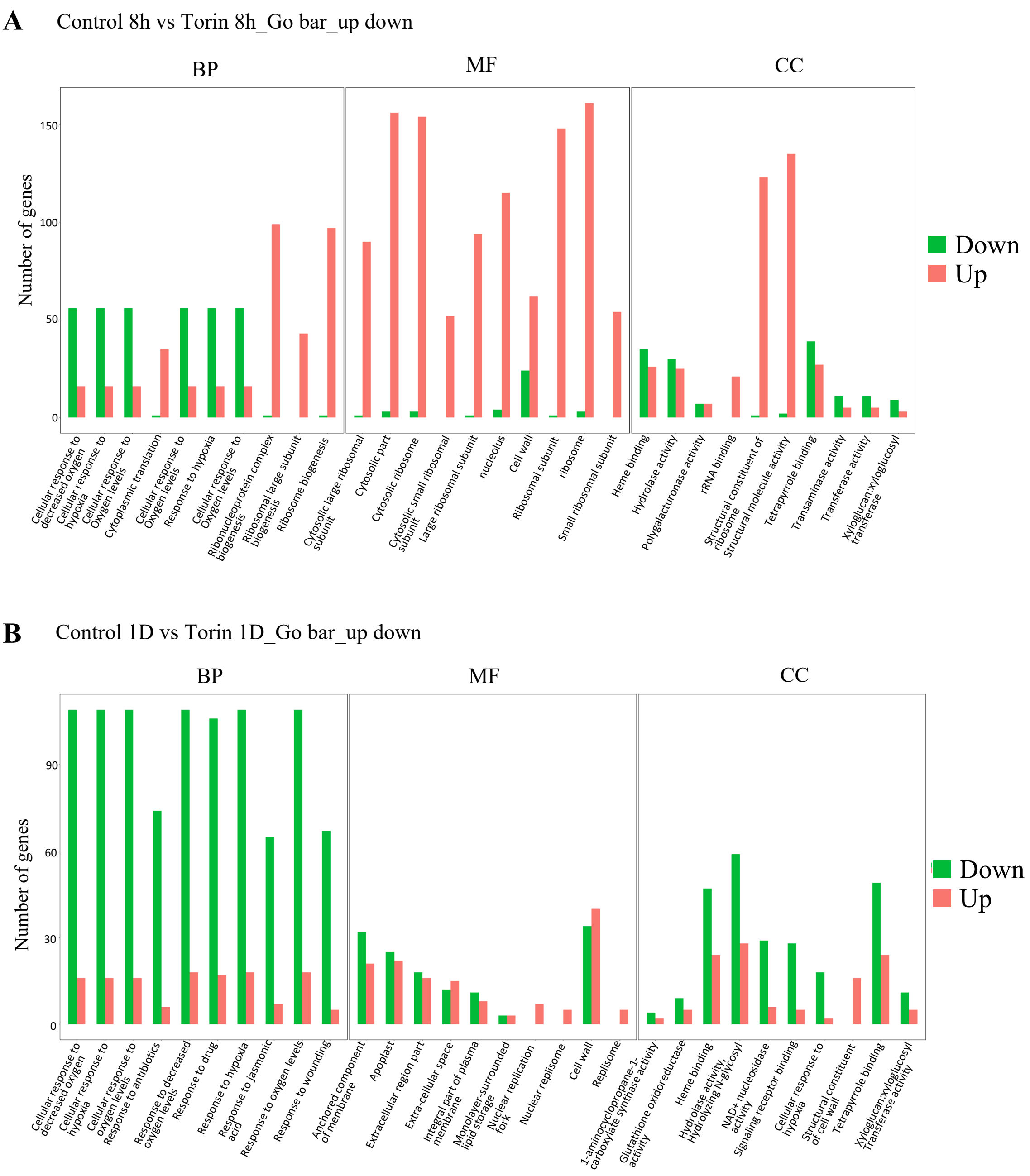

Supplementary Figure S5. Gene Ontology (GO) enrichment analysis

GO enrichment analysis for differentially expressed genes in WT seedling treated with Torin 2 for 8 hours (**A**) or 1 day (**B**). Gene Ontology (GO) annotates genes to biological processes (BP), molecular functions (MF), and cellular components (CC). Green bars represent genes that are downregulated by TOR and red bars represent genes that are upregulated by TOR.

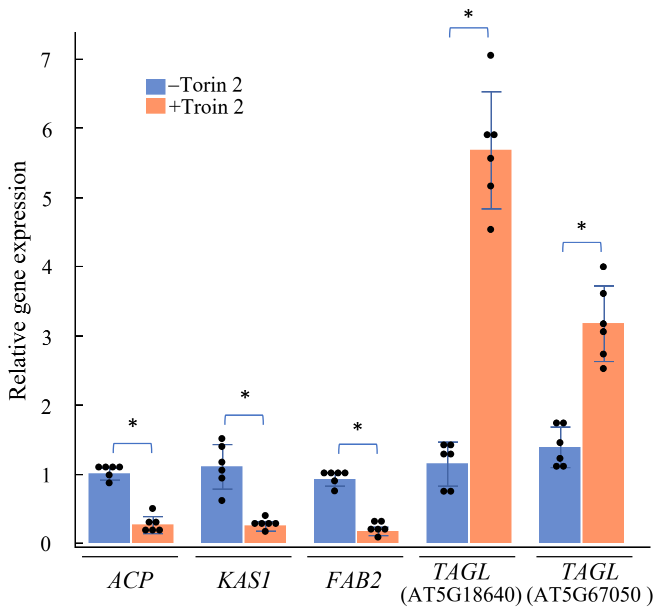

Supplementary Figure S6. qPCR analysis of the selected lipid metabolism genes identified in the differential expression analysis during Torin 2 treatment

Quantitative RT-PCR (RT-qPCR) was performed to assess the expression levels of lipid metabolism genes *TAGL* (AT5G18640), *TAGL* (AT5G67050), *ACP* (AT4G25050), *FAB2*(AT1G43800), and *KAS I* (AT5G46290) in WT seedlings treated with mock (−Torin 2) or Torin 2 (+ Torin 2) for 8 hours. Values are represented as mean ± SD (n = 6) from 6 independent experiments. In each experiment, total RNA was isolated from pooled seedlings for each treatment. Statistically significant differences from the mock treatment were determined by mean crossing point deviation analysis, computed using the relative expression software tool (REST), and are denoted by an asterisk ($P < 0.05$).
